# The Prognosis of Anti-Angiogenesis Treatments Combined with Standard Therapy for Newly Diagnosed Glioblastoma: A Meta-Analysis of Randomized Controlled Trials

**DOI:** 10.1371/journal.pone.0168264

**Published:** 2016-12-22

**Authors:** Yuping Li, Mengzhuo Hou, Guangyu Lu, Natalia Ciccone, Xingdong Wang, Hengzhu Zhang

**Affiliations:** 1 Department of Neurosurgery, The Clinical Medical College of Yangzhou University, Yangzhou, China; 2 Neurosurgical Research, Department of Neurosurgery, Ludwig-Maximilians University of Munich, Munich, Germany; 3 Department of Preventive Medicine, Medical College of Yangzhou University, Yangzhou University, China; 4 Institute of Public Health, Medical School, Ruprecht-Karls-University, Heidelberg, Germany; University of Michigan Medical School, UNITED STATES

## Abstract

**Background and Purpose:**

Although bevacizumab (BV) has been approved as second-line therapy for recurrent glioblastoma (GB), the efficacy and safety of BV for patients with newly diagnosed GB remain unclear.

**Methodology/Principal Findings:**

We systematically searched electronic databases (PubMed, EMBASE, OVID, etc.) to identify related studies published from January 1966 and August 2016. Eight randomized controlled trials including a total of 2,185 patients with GB were included. We found that the median progression-free survival (PFS) was higher in the BV group than in the standard therapy (ST) group (pooled hazard ratio, 0.73; 95%CI, 0.62–0.86; P = 0.0001). Compared with ST, BV improved the PFS rate at 6 months (OR 3.33, 95% CI 2.73–4.06, p<0.00001) and 12 months (OR 2.10, 95% CI 1.74–2.54, p<0.00001). There were no significant differences in median overall survival between the BV and ST groups (OR, 1.01; 95%CI, 0.83–1.23; P = 0.95). The BV group had higher survival rates at 6 months (OR, 1.41; 95% CI, 1.09–1.84; P = 0.01) and 12 months (OR, 1.23; 95% CI, 1.02–1.48; P = 0.03), but a low survival rate at the 36-month follow-up (OR, 0.57; 95% CI, 0.32–0.98; P = 0.04). For the incidence of adverse events, three adverse outcomes were found to be significantly different between BV and ST groups, including hypertension (8.37% vs. 1.62%, p<0.000001), proteinuria (7.65% vs. 0%, p<0.001), and fatigue (14.54% vs. 9.01%, p = 0.05).

**Conclusions/Significance:**

Our study indicates that combination of BV with ST for newly diagnosed GB did not improve the median overall survival but result in longer median PFS, maintaining the quality of life and functional status. However, the long-term use of BV is associated with a higher incidence of adverse events and mortality.

**Study Registration:**

This research was registered at PROSPERO. (Registration Number: CRD42016038247).

## Introduction

Glioblastoma (GB), the most common primary malignant brain tumor in adults, has a dismal prognosis, with a median survival of 14 to 16 months [1]. Even with the best available standard therapies (surgical resection followed by radiotherapy and temozolomide), the prognosis of patients with GB remains low [2,3]. When GB recurs, the median overall survival is typically 3 to 9 months, and available therapies have a limited impact on outcome [4]. Therefore, development of new therapies is essential to improve the overall survival and prognosis of patients with newly diagnosed GB. During the past decade, a large number of targeted therapeutic agents have been developed and evaluated. GB is highly vascular and typically overexpresses vascular endothelial growth factor (VEGF), which promotes tumor angiogenesis, contributing to tumor growth and progression [5–7]. Several clinical trials have suggested that VEGF could be a therapeutic target [8,9]. The U.S. Food and Drug Administration (FDA) approved bevacizumab (BV), a humanized monoclonal antibody to VEGF, for second-line treatment of recurrent GB [10,11]. Despite its prolongation of progression-free survival (PFS) in patients with recurrent GB, the impact of BV on overall survival remains undefined.

Several clinical trials have reported that treatment with combinations of BV and other chemotherapeutic agents results in stable responses and a prolonged 6-month PFS rate in patients with recurrent high-grade glioma, but do not significantly prolong overall survival (OS), compared with previous trials [12–16]. Furthermore, most of the complications caused by the toxicity of the combined chemotherapy led to discontinuation of treatment for patients with GB [17]. In 2009, Zhang et al. conducted a meta-analysis to assess the efficacy and safety of BV alone compared with BV and irinotecan for recurrent GB [18]. The results indicated that the combination of BV and irinotecan may increase the rate of discontinuation and that there was no obvious improvement in overall survival in patients with recurrent GB. Furthermore, this research included only nonrandomized control trials or small-sample retrospective studies. The data from low-quality research results in significant heterogeneity.

From 2009 onward, several randomized controlled trials (RCTs) were conducted to assess the effectiveness of BV for newly diagnosed GB [19–26]. Therefore, it became necessary to conduct a meta-analysis to assess the clinical efficacy of BV compared with standard therapy (ST) or other chemotherapies for newly diagnosed GB and to evaluate the safety and adverse effects of these combinations.

## Materials and Methods

There is no necessary for ethic approval in this meta-analysis, which mainly based on the published studies. This study was conducted according to the Preferred Reporting Items for Systematic Reviews and Meta-Analyses (PRISMA) statement [27].

### Literature Search and Study Selection

Two reviewers (GY.L and MZ.H) performed the literature searching on the BV for patients with newly diagnosed GB to identify relevant articles published between January 1966 and August 2016. Electronic search used “bevacizumab”, “avastin”, “chemotherapy”, “glioblastoma”, “newly diagnosed glioblastoma”, “GB” in Mesh and free terms. We searched PubMed, EMBASE, and the Cochrane Library to identify relevant studies. Manual searches were performed to relevant journal and reference lists of retrieved articles. Two independent reviewers (YP.L and MZ.H) assessed the literature based on the titles and abstracts to identify potentially relevant articles. Full versions of all relevant articles were obtained and inspected.

### Inclusion Criteria and Exclusion Criteria

The inclusion criteria were used for selecting the potential studies: (1) RCTs compare the standard treatment with or without BV for patients with newly diagnosed GB; (2) the patients were adults; (3) the main clinical outcomes and complications were reported; and (4) at least 6 months follow-up.

The exclusion criteria of studies were: (1) review; (2) lack of randomization; (3) data could not be extracted; (4) insufficient clinical data; (5) duplicate papers.

### Data Extraction and Qualitative Assessment

The relevant data from selected studies were independently extracted by 2 reviewers (GY.L and YP.L) as follows: (1) Publishing time, (2) mean age, number of patient, (3) study quality, (4) Follow up, (5) main results, including overall survival (OS) and progression-free survival (PFS), and (6) secondary outcomes, adverse events incidence. Two observers assessed methodological quality of the included studies. The GRADE approach was introduced to evaluate the overall methodological quality of including studies as recommended by Cochrane Handbook for Systematic Review of Interventions [28].

### Statistical Methods

Meta-analysis was performed with RevMan software (version 5.1; The Cochrane Collaboration, The Nordic Cochrane Centre, Copenhagen, Denmark). The hazard ratio (HR) with 95% confidence intervals (CIs) was used to assess main outcomes of the studies, including median OS, median PFS. HRs and their corresponding SEs were directly extracted from studies. If the study did not report a HR but gave the data in the form of the survival curve, survival rates at certain specified times were extracted from them for the reconstruction of the HR estimate and its SEs. The odds ratio (OR) with 95% confidence intervals (CIs) was used to assess the rate of OS and PFS at different follow-ups (6, 12, 24, and 36 months). Statistical significance was accepted as p value less than 0.05. In order, Cochrane Q text (α = 0.05) was performed to analyze the heterogeneity. If the result of the test of the heterogeneity was P > 0.05, the fixed-effect model was used. If the result of heterogeneity test was P < 0.05, then the pooled ORs were analyzed using the random effects model. Publication bias was evaluated with Egger’s regression test in which P value less than 0.10 were considered representative of statistically significant publication bias.

## Results

### Description of the Studies

**Fig 1:**
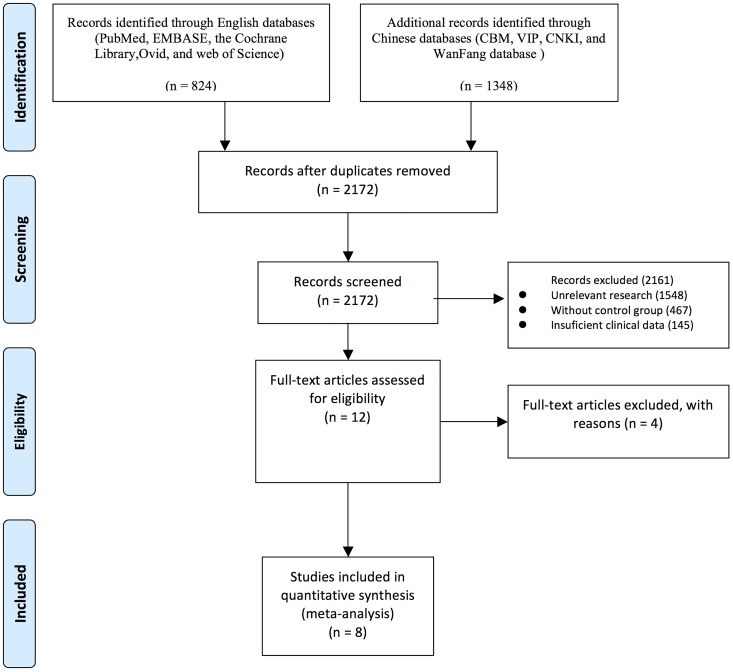
The PRISMA flow chart of the meta-analysis.

Fig 1 is a flowchart describing the study selection and inclusion process. The primary search yielded 2,172 potentially relevant articles (Fig 1). Of these, 2161 were excluded after reading titles and abstracts. Two independent reviewers (Y.P.L and H.Z.Z) then reviewed the full texts of the remaining 12 articles. Four more studies were excluded. Finally, we identified 8 RCTs including a total of 2,185 patients with newly diagnosed GB (Table 1) [19–26]. Sample size ranged from 54 to 921. Six studies were published in English [19–24] and two in Chinese [25,26]. These articles were published between 2011 and 2015. Details of the treatment and functional outcome measures are summarized in Table 1.

**Table 1:** Characteristics of included studies.

Study	Inclusion criteria	Age (y)		Cases (n/M)		Treatment		Outcome	Side-effect	Follow-up (m)
		Experiment	Control	Experiment	Control	Experiment	Control			
Albert Lai 2011	age > 18 years	57.4	59.4	70/39	110/70	BE	RT	OS	Y	42
	newly diagnosed GB					RT	TMZ	PFS		
	KPS >60					TMZ		Toxicity&Safety		
MR. Gilbert 2014	age > 18 years	-	-	312	309	BE	RT	OS	Y	30
	newly diagnosed GB					RT	TMZ	PFS		
	KPS>70					TMZ		Toxicity&Safety		
								MGMT status		
OL. Chinot 2014	age > 18 years	57	56	458	463	BE	RT	OS	Y	32
	newly diagnosed GB					RT	TMZ	PFS		
						TMZ		KPS		
	supratentorial							GHS		
B. Chauffert 2014	ages from 18 and 70 y	60.2	60.9	60/34	60/37	BE	RT	OS	Y	24
	KPS>50					RT	TMZ	PFS		
	histologically confirmed					TMZ		OS		
						IRI				
U Herrlinger 2013	newly diagnosed GB	56	56	116/80	54/34	BE	RT	OS		36
	KPS>70					RT	TMZ	PFS		
	age > 18 years					TMZ		QoL		
						IRI				
JA. Carlson 2015	age > 18 years	55.9	59.5	30/17	26/16	hypo-IMRT	hypo-IMRT	OS	Y	60
	newly diagnosed GB					BE	TMZ	PFS		
	KPS>60					TMZ				
KF. Hofland 2014	age > 18 years	62	59	32/21	31/18	BE	IRI	Response Rate	Y	31
	newly diagnosed GB					RT	RT	OS		
						TMZ	TMZ	PFS		
Jiaqi Wang 2013	newly diagnosed GB	53.6	54.7	27/16	27/14	BE	RT	RECIST	Y	24
	KPS>60					RT	TMZ	OS		
						TMZ				

GB: Glioblastoma; KPS: Karnofsky performance status; RT: radiotherapy; TMZ: temozolomide; BV: bevacizumab; OS: overall survival; PFS: progression-free survival; GHS: GlobalHealthStatus; GoL:quality of life; Y: yes;

### The Median PFS and PFS Rate

**Fig 2:**
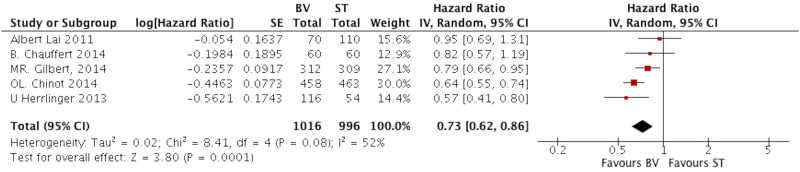
The pooled HR of median PFS comparing BV with ST in patients with GB.

Five studies presented the data of median PFS comparing BV with ST group [19,20,22–24]. Since the significant difference was observed in heterogeneity test (I^2^ = 52, P = 0.08), the random-effects model was applied to analyze data. The pooled HR was 0.73 (95%CI, 0.62–0.86; P = 0.0001), as shown in Fig 2.

**Fig 3:**
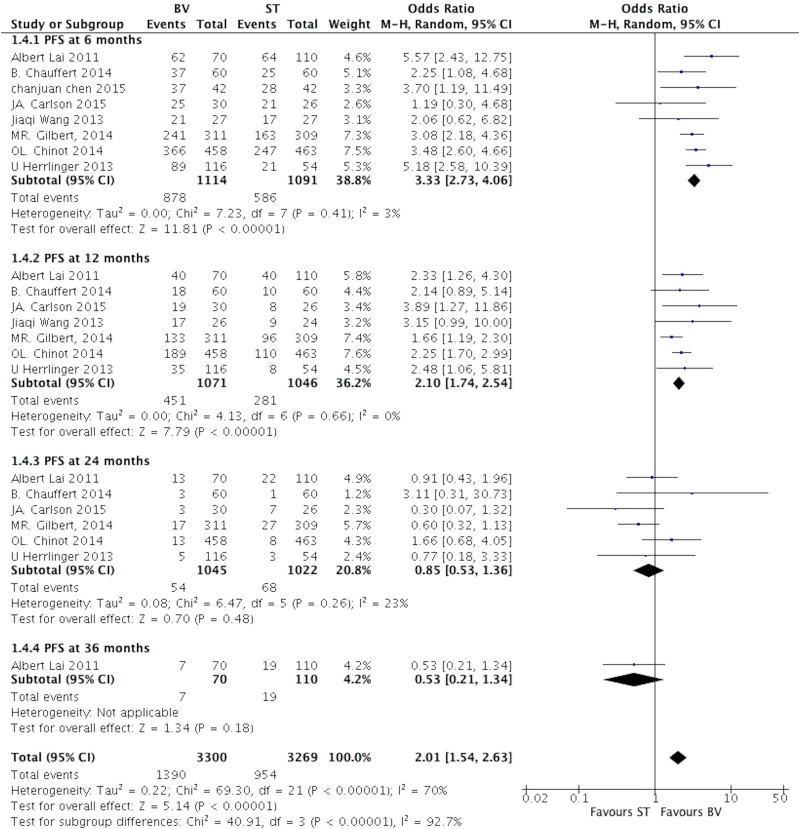
The pooled analysis of PFS rate comparing BV with ST in patients with GB at different follow-ups.

Eight studies reported the PFS rate at different follow-ups comparing BV with ST group [19–26]. No statistically significant heterogeneity was observed between studies, I^2^ = 3% at 6 months, 0% at 12 months, and 23% at 24 months; so the fixed effect model was applied. The pooled OR of PFS was 3.33 at 6 months (95%CI, 2.73–4.06; P<0.00001), 2.10 at 12 months (95%CI, 1.74–2.54; P<0.00001), 0.85 at 24 months (95%CI, 0.53–1.36; P = 0.48), and 0.53at 36 months follow-ups (95%CI, 0.21–1.34; P = 0.18). (Fig 3)

### The Median OS and Survival Rate

**Fig 4:**
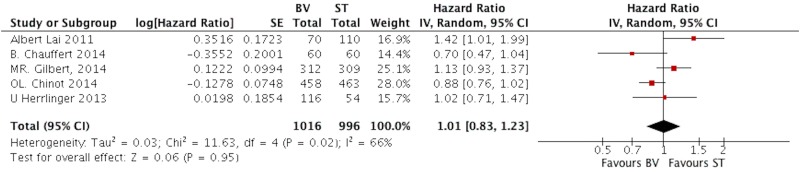
The pooled HR of median OS comparing BV with ST in patients with GB.

Five studies presented the data of median OS [19,20,22–24]. The heterogeneity test showed significant differences in each study (I2 = 66, P = 0.02). Then we applied the random-effects model to analysis the data. The pooled HR of median OS was 1.01 (95%CI, 0.83–1.23; P = 0.95) as shown in Fig 4.

**Fig 5:**
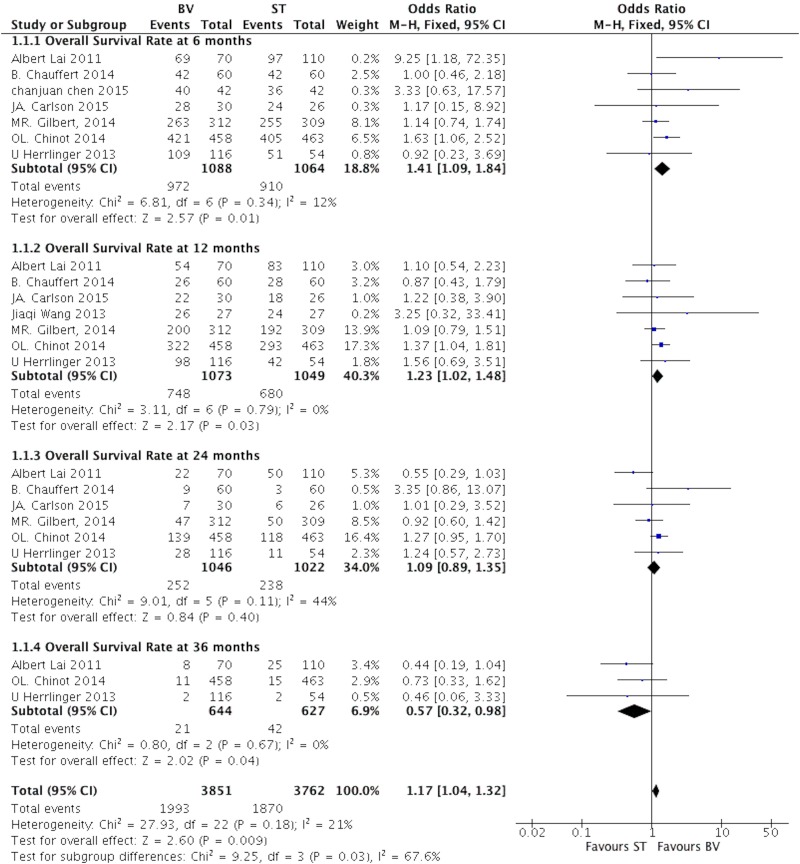
The pooled analysis of OS rate comparing BV with ST in patients with GB at different follow-ups.

All included studies [19–26] reported the data of survival rate in different follow-up end points. The test of total heterogeneity showed no significant differences (I^2^ = 21, P = 0.18). The fixed-effects model was applied. The pooled OR of survival rate was 1.41 (95% CI, 1.09–1.84; P = 0.01) at 6 month, 1.23 (95% CI, 1.02–1.48; P = 0.03) at 12 month, 1.09 (95% CI, 0.89–1.35; P = 0.40) at 24 month. Moreover, we noted that low survival rate in BV group at 36 month, and the OR was 0.57 (95% CI, 0.32–0.98; P = 0.04). (Fig 5)

### Safety of BV for Patients with Newly Diagnosed GB

**Fig 6:**
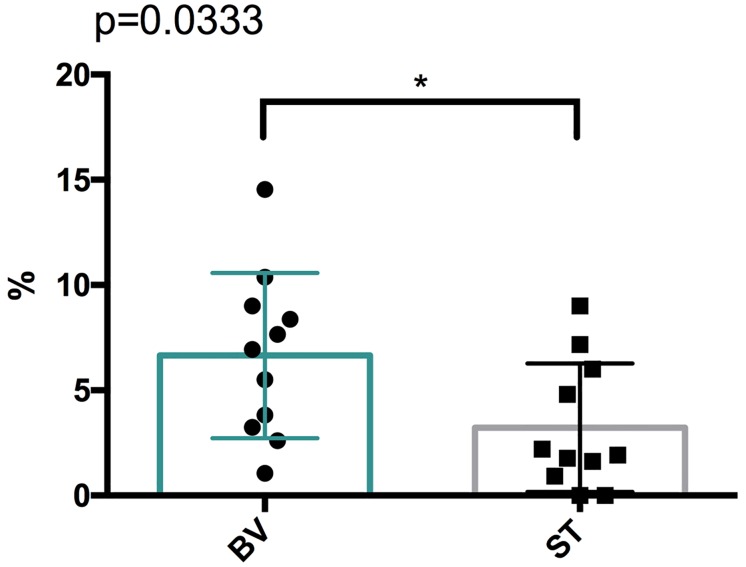
The incidence of all severe AEs in patients with newly diagnosed GB.

Of included trials, five trials provided data of AEs incidence [19,20,22–24]. From these events, three adverse outcomes were found to be significantly different between bevacizumab and standard treatment groups, including hypertension (8.37% vs. 1.62%, p<0.000001), proteinuria (7.65% vs. 0%, p<0.001), fatigue (14.54% vs. 9.01%, p = 0.05), as shown in Table 2. The incidence of all severe AEs also showed significant between BV and ST group, as showed in Fig 6. There was significant trend toward with regard to BV therapy and high severe AEs incidence (P = 0.033).

**Table 2:** The adverse events of patients with newly diagnosed GB between BV and ST groups.

Adverse Event	n (studies)	BV group		ST group		OR	P
		n(III-IV)	n(total)	n(III-IV)	n(total)		
Cerebral ischemia	2	7	127	1	56	3.21 [0.39, 26.71]	0.28
Cerebral Hemorrhage	4	9	848	7	750	2.30 [0.96, 5.54]	0.06
Diarrhea	3	5	154	0	83	6.14 [0.34, 112.49]	0.22
Neutropenia	3	31	344	19	316	1.55 [0.86, 2.80]	0.15
Nausea and vomit	2	11	287	5	260	2.03 [0.70, 5.93]	0.19
Fatigue	2	48	330	21	233	1.72 [1.00, 2.96]	0.05
Hypertension	4	71	848	12	739	5.54 [2.98, 10.29]	<0.000001
Infection	3	16	154	4	83	2.29 [0.74, 7.09]	0.15
Proteinuria	2	33	431	0	560	94.24 [5.76, 1542.53]	<0.001
Thromboembolic event	4	56	805	55	766	0.97 [0.66, 1.42]	0.86
Anemia	3	9	344	7	317	1.19 [0.44, 3.23]	0.73

### Qualitative Assessment and Publication Bias

**Fig 7:**
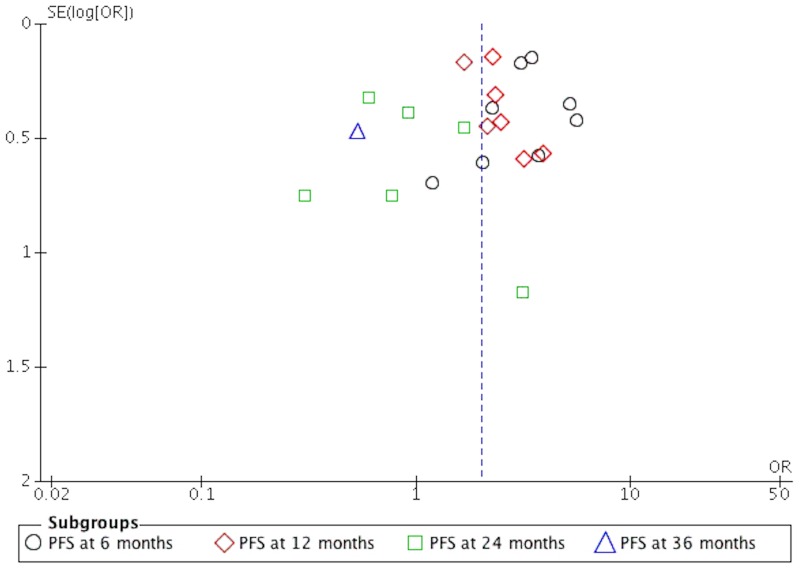
The funnel plot of PFS on patients with newly diagnosed GB.

**Fig 8:**
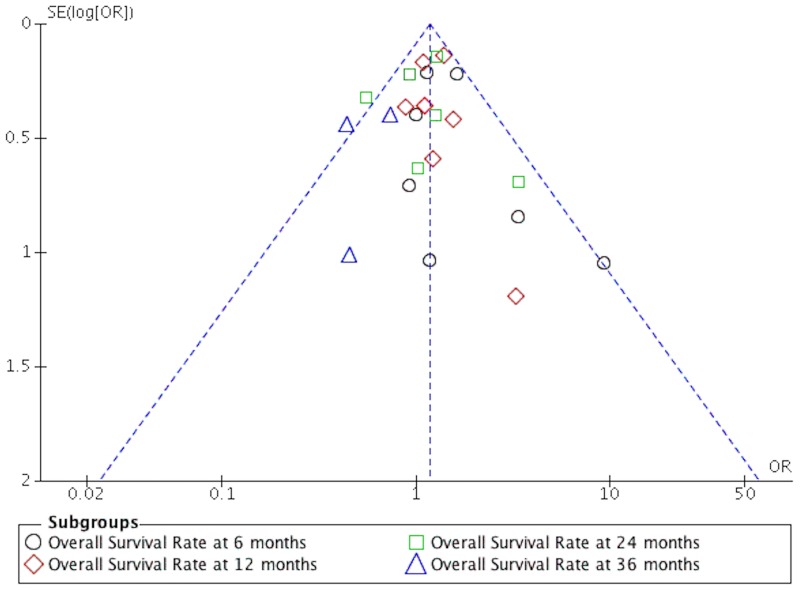
The funnel plot of OS on patients with newly diagnosed GB.

The quality of the studies included in this meta-analysis is shown in Table 1. It can be seen from the funnel plot that the publication bias was low to moderate regarding PFS and OS (Figs 7 and 8).

## Discussion

GB is an aggressive malignant brain tumor with a poor prognosis. Despite the use of some anti-angiogenesis treatments, there remains no successful therapy [1,2]. The average survival time is only 14 months after diagnosis [3]. A major clinical problem is the extent to which the glioma cells invade adjacent brain tissue, making complete surgical removal impossible [29]. Some research has indicated that GB has multistep tumorigenesis ability to activate the vascularization of brain tissue to form new blood vessels, which contribute to tumor growth and proliferation [29,30]. VEGF has shown promise as a therapeutic target in the treatment of brain tumors [31]. Bevacizumab was developed to directly inhibit VEGF-associated angiogenic effects by blocking activation of the VEGF receptor (VEGF-R) [32,33]. Recently, BV was used as second-line therapy for recurrent GB in some clinical trials [34–37]. To date, no meta-analysis has investigated the effects of BV on the prognosis of newly diagnosed GB.

The principal findings of our meta-analysis can be summarized as follows: (1) BV combined with standard treatments could improve the median PFS of patients with newly diagnosed GB and the PFS rate in short-term follow-up (6 and 12 months). (2) There is no significant difference in median overall survival between patients treated with BV and those treated with ST; however, more survivors were observed in the BV group in early follow-up (<12), with this trend reversing at 36 months. (3) The incidence of severe adverse effects was higher after BV therapy than after ST.

Our results indicate that BV could prolong the median PFS of patients with newly diagnosed GB as compared with a ST group. More patients had attained PFS in the BV group than in the ST group at 6 months (78.26% vs. 53.19%) and 12 months (41.26% vs. 27.34%) follow-up; at the 2-year follow-up, we found the PFS rates of the BV and ST groups were equivalent. Furthermore, one study reported a decreased PFS rate in the BV group compared with the ST group at 3 years. On the basis of the aforementioned evidence, we conclude that BV improves the short-term PFS rate (<12 months). These results are consistent with previous clinical trials. The AVAglio trial reported median PFS durations of 10.6 months in the BV group and 6.2 months in the ST group [23], and the RTOG 0825 trial obtained the same results for PFS duration (10.7 months in the BV group vs. 7.3 months in the ST group) [22]. However, these two large RCTs did not report PFS rates at 3 years of follow-up. The decreased PFS was observed after long-term application of BV (>3 years), which might be caused by BV resistance, chronic comorbid conditions, or side effects. Additional clinical trials are needed to clarify this issue.

Our study indicated that BV did not improve the median overall survival of patients with newly diagnosed GB. This finding coincides with those of the AVAglio, RTOG 082512, and GLARIUS trials, indicating approximately equivalent overall survival (OS) for the BV and ST groups (11.1% vs. 11.7%, p = 0.59) [22–24]. However, comparison of OS rates at different follow-up points revealed more survivors in the BV group early in follow-up (<12 months), with this trend reversing at 36 months. Our data indicate that with prolonged use of BV, patients with GB exhibited worse neurocognition and higher mortality, compared with controls.

Until now, our knowledge of the mechanism underlying the pro-tumor effect of long-term BV treatment has been limited. One clinical study reported that nearly half of patients with GB treated with BV had a low response rate [38]. The rebound of tumor invasion and metastasis was observed by radiography [39]. Several experiments in animals have found more perivascular invasion, more peritumoral satellite lesions, and higher expression of invasion-related proteins with long-term BV treatment as compared with short-term BV treatment [40,41]. Lucio Eterovic et al. found that with increasing concentration of BV administered, U87 glioma cells secreted greater amounts of matrix metalloproteinase (MMP)-2, MMP-9, and MMP-12, and activated other angiogenic pathways, promoting the migration and invasion of tumor cells [40]. More importantly, in this research, BV was combined with MMP inhibitor (GM6001) in the glioma mouse model. The results indicated that those therapies could prolong survival and suppress tumor progression [40]. Recently, results from animal research suggested that minocycline reduces glioma growth by inducing glioma autophagy [42]. In addition, an ongoing clinical trial found that use of radiation, followed by BV and minocycline, to treat recurrent GB may improve the effect of long-term BV treatment. (ClinicalTrials.gov Identifier: NCT01580969)

Drug resistance was considered another factor influencing the effectiveness of BV treatment for GB. BV was able to inhibit the VEGF—VEGFR signal pathway to suppress tumor growth [43]. Although this could reduce cerebral oxygen delivery, which up regulates the expression of HIF-10, it will result in a rebound in the expression of VEGF genes [44,45]. In addition, a multidirectional cytokine, placental growth factor (PlGF), which is homologous to VEGF, can promote the proliferation and migration of endothelial cells. When VEGF is inhibited, endothelial cells upregulate the expression of PlGF to maintain tumor-associated vascular growth [46]. Recently, several researchers found that some cytokines overexpressed in glioma cells after BV treatment contribute to angiogenesis, including platelet-derived growth factor, fibroblast growth factor, interleukin-8 and -10, and angiopoietin-1 [47–49].

The adverse events of BV might affect the quality of life and prognosis of patients with GB. The most common adverse events of BV included abdominal pain, headache, fatigue, hypertension, diarrhea, neutropenia, wound infections, cerebral hemorrhage or ischemia, nausea and vomiting, thromboembolism, and anemia. To assess the incidence of severe adverse effects (AEs) in BV treatment of adults newly diagnosed with GB, we extracted all BV-associated severe AEs from the five studies included in this meta-analysis. We found that those treated with BV had an increased symptom burden and incidence of severe AEs. In a previous meta-analysis assessing the risk of adverse vascular events in patients newly diagnosed with GB, BV therapy was not found to significantly influence the risk of all-cause discontinuation, thrombocytopenia, deep vein thrombosis, and pulmonary embolism in adult patients newly diagnosed with GB [50]. The authors, however, did report a trend toward significance with respect to BV therapy and the risk of pulmonary embolism [50]. Therefore, in patients under treatment with BV, care should be taken to monitor blood pressure, blood clotting function, kidney function, and other indicators.

## Limitation

The present study has several limitations. First, because only eight RCTs were included, small sample size and highly selected patient populations could generate selective bias. Second, there were insufficient data on mutation status and BV resistance. Third, most of the studies included studies reported the OS and PFS rates only within 2 years of follow-up. Considering the long-term survival rate, further studies reporting OS rates at 3 years or longer follow-ups are needed to clarify this issue. Fourth, although all the included studies used ST combined with BV for newly diagnosed GB, two studies administered BV combined with irinotecan and one study combined BV with hypofractionated intensity modulated radiation therapy in the experimental groups, which may have caused heterogeneity in comparisons. Fifth, new therapies are continuing to show the potential benefits for patients with newly diagnosed GB, such as carmustine wafer (CW). A meta-analysis from Chowdhary SA et al. indicated that CW treatment could significantly improve the overall several and survival rate of patients with newly diagnosed or recurrent high-grade GB [51]. Therefore, further studies are required to investigate the effect of BV combined with other therapies for newly diagnosed GB, such as CW. Sixth, relevant factors could influence the prognosis of patients with newly diagnosed GB, including patient age, gender, obesity, steroid use, and smoking history. Because such individual data were lacking, this study did not assess these factors.

## Conclusions

In summary, our study indicates that BV therapy does not appear to improve median OS in patients with newly diagnosed GB, whereas it prolongs median PFS. We also found that BV therapy improves the PFS and OS rates at the 6- and 12-months follow-ups. However, at 36 months, the decreased OS rate and high incidence of severe AEs in those treated with BV make quality of life an issue.

## Supporting Information

S1 Checklist PRISMA Checklist. (DOC)
